# Rational Construction of a Mitochondria-Targeted Reversible Fluorescent Probe with Intramolecular FRET for Ratiometric Monitoring Sulfur Dioxide and Formaldehyde

**DOI:** 10.3390/bios12090715

**Published:** 2022-09-03

**Authors:** Jinxiao Lyu, Chunfei Wang, Xuanjun Zhang

**Affiliations:** 1 Cancer Centre and Centre of Reproduction, Development and Aging, Faculty of Health Sciences, University of Macau, Macau SAR 999078, China; 2 MOE Frontiers Science Center for Precision Oncology, University of Macau, Macau SAR 999078, China

**Keywords:** reversible probe, mitochondria, sulfur dioxide, formaldehyde, ratiometric sensing

## Abstract

Sulfur dioxide (SO_2_) and formaldehyde (FA) are important species that maintain redox homeostasis in life and are closely related to many physiological and pathological processes. Therefore, it is of great significance to realize the reversible monitoring of them at the intracellular level. Here, we synthesized a reversible ratiometric fluorescent probe through a reasonable design, which can sensitively monitor SO_2_ derivatives and FA, and the detection limit can reach 0.16 μM. The probe can specifically target mitochondria and successfully monitor the fluctuations of SO_2_ and FA in living cells. It also works well in the detection of SO_2_ and FA in zebrafish. This high-performance probe is expected to find broad in vitro and in vivo applications.

## 1. Introduction

In order to maintain life activities, organisms undergo redox reactions all the time, in which multiple substances jointly maintain redox homeostasis [1,2,3]. Among them, sulfur dioxide (SO_2_) is an important species for maintaining the redox balance in organisms and plays an important role in many bioactivities, including regulating vascular function [4] and protecting against acute lung injury [5]. It is also considered to be the fourth possible gas messenger molecule after nitric oxide [6], hydrogen sulfide [7,8] and carbon monoxide [9,10]. SO_2_ can be inhaled from the environment and can be synthesized endogenously through an enzymatic reaction in vivo. Excessive SO_2_ may lead to respiratory stimulation and a variety of diseases [11]. However, SO_2_ in physiological concentrations can not only resist oxidative stress, but also regulate blood pressure and lipid metabolism [12]. Formaldehyde (FA) is another biological information molecule, which is widely present in most organisms, including humans, and is one of the important metabolic byproducts produced through physiological activities [13,14]. FA produced by endogenous sources include methanol metabolism, amino acid metabolism, lipid peroxidation and the demethylation of nucleic acids and histones [15,16]. It is also an important metabolic intermediate coexisting in almost all cells [17] and can be used for the biosynthesis of purine and some other alkaloids [18]. The degradation of FA is mainly mediated by formaldehyde dehydrogenase (FDH) and alcohol dehydrogenase 1 (ADH1) [19], which rely on glutathione (GSH), which is also closely related to the metabolism of SO_2_. In summary, FA can not only oxidize SO_2_ derivatives directly, but also antagonize the signal transduction process mediated by SO_2_. Therefore, the real-time monitoring of SO_2_ and FA levels in organisms has important biological significance.

In recent years, researchers have established a variety of methods to detect SO_2_ and FA. Among them, fluorescent probes are widely used in the study of various life phenomena in cells [20,21,22] and in vivo due to their advantages of low damage, high specificity, high sensitivity and real-time dynamic imaging [23]. In previous studies, benzopyranium has been proven to be an efficient structure to detect sulfur dioxide [24,25], and its recyclable and reversible detection with some oxidizing species may yet be realized [26,27,28]. In this paper, we designed and synthesized a fluorescent B2P probe with benzopyranium as the detection site, which can reversibly detect SO_2_ and FA. In order to improve sensitivity and realize ratiometric sensing, a Förster resonance energy transfer (FRET) mechanism is usually adopted in the design of probes [29]. Due to its high fluorescence quantum yield, good photostability and convenient modification [30,31], BODIPY was selected as an energy donor in the probe. The acceptor and donor parts of the probe were connected with a triazole formed through a click reaction. As expected, the benzopyrylium moiety in the probe could efficiently react with SO_2_, resulting in an increase in green fluorescence and a disappearance of red fluorescence emissions. Moreover, the adducts were reversibly restored through the oxidation of FA and realized the recovery of the red emissions. Furthermore, the probe was also applied for the imaging of SO_2_ and FA in living cells and zebrafish. At the same time, considering that mitochondria are the redox metabolism centers of cells [32], it was necessary to make the probe have the ability to target mitochondria. Therefore, a structure with double cations was introduced to the probe B2P. The double cation structure (benzopyrylium and quaternary ammonium) could ensure the accumulation of the probes in mitochondria through the electrostatic force between the positive charges and mitochondrial membrane potential [33,34,35]. Moreover, quaternary ammonium cations that did not participate in the detection reaction could enable the probe to be located in mitochondria even after forming adducts with HSO_3_^−^. Therefore, the probe could potentially be a useful tool for studying the metabolism of SO_2_ and FA in complex living systems.

## 2. Materials and Methods

### 2.1. Synthesis of the Probe

#### 2.1.1. Synthesis of Compound **2**

Compound **4** was synthesized via the substitution reaction of compounds **5** and **6**: Compound **6** (516 mg, 3 mmol, 1 equiv) was dissolved in 20 mL acetonitrile and was added into a dry flask with a stir bar before 780 mg of DIPEA (6 mmol, 2 equiv) and 2.7 g compound **5** solution in Tol (80 wt%) were added. Then, the reaction was stirred at 60 °C under a nitrogen atmosphere for 48 h. The solvent was removed under reduced pressure and the residue was purified with silica column chromatography (DCM:MeOH, 10:1). Product: 336 mg (yield 31%).

To a dry flask with a stir bar, 370 mg of compound **4** (1.8 mmol, 1 equiv) and 520 mg of 4-(diethylamino) salicylaldehyde (2.7 mmol, 1.5 equiv) were added. Then, 5 mL of concentrated H_2_SO_4_ was added dropwise before the mixture was heated to 90 °C. The reaction was stirred under a N_2_ atmosphere for 12 h and quenched by adding 1 mL of HClO_4_ (71%, 12.5 M) and 50 mL of H_2_O. Then, the suspension was filtered, and the filter residue was collected and washed with water. Product: 710 mg (yield 64%). The ^1^H NMR (Figure S7, 400 MHz, acetone-d6) δ 8.69 (d, J = 8.2 Hz, 1H), 8.37–8.29 (m, 2H), 7.98 (dd, J = 18.4, 8.9 Hz, 2H), 7.48 (dd, J = 9.3, 2.5 Hz, 1H), 7.39 (d, J = 2.4 Hz, 1H), 7.35–7.30 (m, 2H), 4.90 (d, J = 2.5 Hz, 4H), 4.18 (dd, J = 6.4, 4.1 Hz, 4H), 4.12 (dd, J = 6.3, 4.2 Hz, 4H), 3.82 (q, J = 7.1 Hz, 4H), 3.74 (t, J = 2.5 Hz, 2H), 1.36 (t, J = 7.1 Hz, 6H). The ^13^C NMR (Figure S8, 101 MHz, DMSO) δ 167.12, 158.99, 155.87, 148.90, 132.49, 130.89, 119.07, 117.67, 114.88, 114.11, 109.65, 108.74, 96.52, 96.43, 84.60, 71.59, 71.52, 56.86, 50.01, 49.07, 45.80, 12.92.

#### 2.1.2. Synthesis of Compound **3**

Compound **7** was synthesized from compound **9** with the reported method [36]. To a dry flask with a stir bar, a solution of 250 mg of compound **7** (1.31 mmol) in 100 mL of THF with 1mM TFA was added before 0.27 mL of 2,4-Dimethyl-1H-pyrrole (2.62 mmol, 2 equiv) was added. Then, the mixture was stirred at room temperature for 12 h. After TLC monitoring, 322 mg of p-chloranil (1.31 mmol, 1 equiv) was added to the flask, and the mixture was stirred at room temperature for 2 h. Then, 1 mL of Et_3_N and 0.83 mL of BF_3_·OEt_2_ were added dropwise. The mixture was further stirred overnight. After TLC monitoring, the reaction was quenched by adding water, and the product was extracted through DCM and washed with water (3 × 100 mL). The organic layer was dried over anhydrous sodium sulfate, and the solvents were removed under reduced pressure. The residue was purified with silica column chromatography (Hex: DCM, 5:1). Product: 285 mg (Yield 53%). The ^1^H NMR (Figure S9, 400 MHz, chloroform-d) δ 7.24–7.19 (m, 2H), 7.08–7.03 (m, 2H), 6.00 (s, 2H), 4.23 (t, J = 5.0 Hz, 2H), 3.69 (t, J = 4.9 Hz, 2H), 2.57 (s, 6H), 1.45 (s, 6H). The ^13^C NMR (Figure S10, 101 MHz, CDCl3) δ 158.77, 155.36, 143.13, 141.52, 131.78, 129.35, 127.78, 121.19, 121.16, 115.13, 66.95, 50.23, 14.62, 14.60, 14.58.

#### 2.1.3. Synthesis of the Probe

**Scheme 1:**
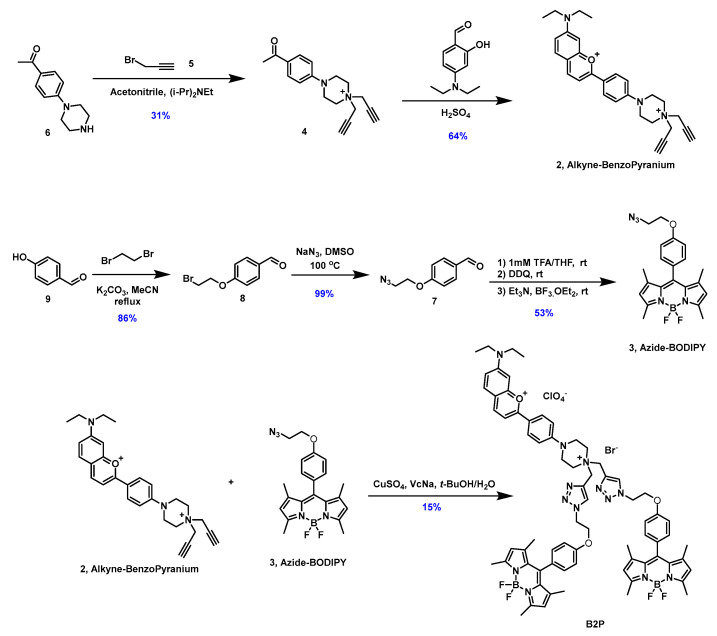
The synthesis route of the probe B2P.

The synthesis of the probe is shown in Scheme 1. The probe was synthesized through a click reaction: To a dry flask with a stir bar, 49.5 mg of compound **2** (0.08 mmol) and 65.5 mg of compound **3** (0.16 mmol, 2 equiv) dissolved in 10 mL *t*-BuOH were added. Then, 16 mg of CuSO_4_·5H_2_O (0.064 mmol, 0.8 equiv) and 25 mg of sodium ascorbate (0.128 mmol, 1.6 equiv) dissolved in 10 mL H_2_O were added into the reaction. The system was stirred at room temperature under a N_2_ atmosphere for 24 h. After TLC monitoring, the reaction product was extracted with 5 mL DCM 3 times. The organic layers were washed with a saturated NaCl aqueous solution and dried over anhydrous sodium sulfate. The solvents were removed under reduced pressure. Then, the residue was purified with silica column chromatography (DCM:MeOH, 15:1). Product: 17 mg (yield 15%). The ^1^H NMR (Figure S5, 400 MHz, acetone-d6) δ 8.85 (s, 2H), 8.70 (d, J = 8.2 Hz, 1H), 8.32 (d, J = 9.3 Hz, 2H), 8.02 (d, J = 9.4 Hz, 1H), 7.94 (d, J = 8.3 Hz, 1H), 7.51 (dd, J = 9.4, 2.4 Hz, 1H), 7.42–7.39 (m, 1H), 7.34 (d, J = 9.3 Hz, 2H), 7.27–7.24 (m, 4H), 7.19–7.16 (m, 4H), 6.07 (s, 4H), 5.10–5.05 (m, 8H), 4.66 (t, J = 5.0 Hz, 4H), 4.27 (d, J = 5.6 Hz, 4H), 3.91 (t, J = 5.0 Hz, 4H), 3.84 (q, J = 7.2 Hz, 4H), 2.84 (s, 12H), 2.47 (s, 12H), 1.37 (t, J = 7.1 Hz, 6H). The ^13^C NMR (Figure S6, 101 MHz, DMSO) δ 158.91, 158.84, 155.82, 155.13, 148.79, 143.08, 142.26, 134.95, 132.49, 131.46, 130.85, 130.29, 130.10, 129.69, 127.05, 121.71, 115.71, 114.66, 96.39, 66.74, 66.62, 64.23, 53.31, 49.85, 45.77, 29.28, 25.56, 22.55, 14.64, 14.43, 12.91. HRMS(Figure S16): calculated m/z 1257.64 (z = 1), 628.82 (z = 2); found: 1257.7894, 628.4760.

### 2.2. Fluorescence Spectra Measurements

The probe B2P was dissolved in acetonitrile and prepared into a 1 mM stock solution, and was further diluted to 10 μM in 10 mM PBS buffer (pH 7.4, containing 20% CH_3_CN). All analytes were prepared with deionized water. The spectral experiment was carried out with a 1 cm standard quartz cell at room temperature.

### 2.3. Cell Culture and Imaging

HeLa cells were cultured in a humidified environment with 5% carbon dioxide at 37 °C. Before the confocal imaging experiment, the cells were implanted onto a 35 mm confocal dish and adhered for 24 h. Before imaging, the cells were washed three times with phosphate-buffered saline.

### 2.4. Living Zebrafish Imaging

All larval zebrafish were provided by the animal facility of the University of Macau. All experiments were conducted in full compliance with international ethical guidelines and approved by the institutional committee. Zebrafish embryos were cultured in an E3 medium supplemented with PTU. Before the imaging experiment, 5-day-old zebrafish were fed with a 20 μM probe and cultured for 30 min. Then, the zebrafish were treated with NaHSO_3_ (100 μM) for 30 min and FA (500 μM) for 60 min. After the culture, zebrafish larvae were anesthetized with MS222, and fluorescence imaging was performed under a laser confocal microscope.

## 3. Results and Discussion

### 3.1. FRET Effect between the Acceptor Benzopyrylium and Donor BODIPY

**Figure 1:**
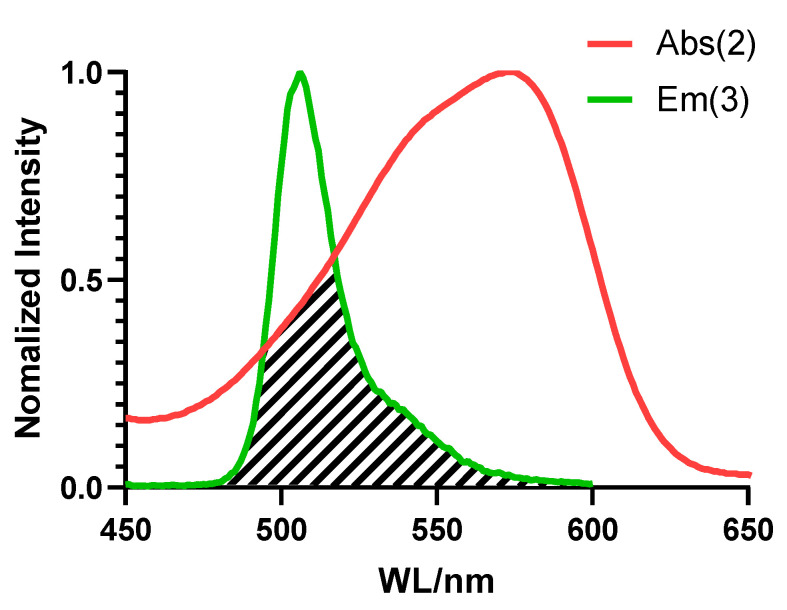
Necessary conditions for FRET to occur: The normalized fluorescence spectrum of the donor part (BODIPY, compound **3**, shown as the green curve) overlaps the normalized absorption spectrum of the acceptor part (benzopyrylium, compound **2**, shown as the red curve).

**Figure 2:**
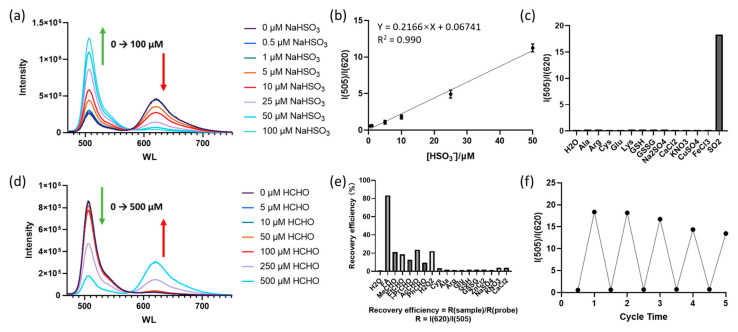
(**a**) Fluorescence spectra of probe B2P (10 μM) in the presence of different concentrations of NaHSO_3_; (**b**) the relationship between the fluorescence ratio (I_505_/I_620_) and the concentration of NaHSO_3_; (**c**) fluorescence ratio (I_505_/I_620_) of probe B2P in the presence of different analytes (200 μM); (**d**) fluorescence spectra of the B2P–HSO_3_ adduct (10 μM probe and 100 μM NaHSO_3_) in the presence of different concentrations of FA; (**e**) the recovery efficiency of the fluorescence ratio (I_620_/I_505_) of probe B2P–HSO_3_ in the presence of different aldehydes, oxidizing substances and other analytes (400 μM); (**f**) the fluorescence ratio (I_505_/I_620_) in the reversible cycle of B2P under alternate additions of NaHSO_3_ and FA.

To facilitate an efficient FRET, an overlap between the emission spectrum of the donor fluorophore and the absorption spectrum of the acceptor fluorophore was required [37]. As shown in Figure 1, the green curve represents the normalized fluorescence spectrum of the BODIPY part, while the red curve represents the normalized absorption spectrum of benzopyrylium. The shaded area showed an obvious overlap, which met the necessary requirement of at least a 30% overlap for an effective FRET [38]. For more spectral properties of the compounds, see the Supplementary Material (Figures S2–S4). At the same time, as shown in Figure 2a, after excitation, the B2P molecule emitted the red fluorescence of the acceptor. Moreover, after the fluorescence of the acceptor was quenched with NaHSO_3_, the fluorescence emission of the donor was also significantly enhanced.

### 3.2. Fluorescence Spectra of B2P for Sulfur Dioxide Monitoring

**Scheme 2:**
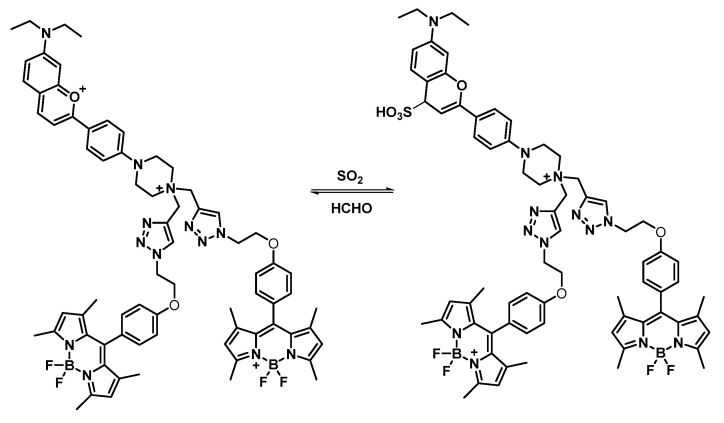
Sensing mechanism of the probe.

The sensing performance of the probe for sulfur dioxide was investigated by measuring the fluorescence spectra. The response mechanism of the probe for detecting sulfur dioxide is shown in Scheme 2. SO_2_ was reversibly added to the benzopyranium site [25] and caused fluorescence quenching. The probe showed an obvious ratiometric fluorescence response to SO_2_ under a single excitation, with two emission bands. As shown in Figure 2a, the fluorescence peaked at 620 nm and decreased with the addition of NaHSO_3_, and the emission at 505 nm increased gradually, which was attributed to the formation of the adduct between HSO_3_^-^ and B2P. More importantly, the fluorescence intensity ratio (I_505_/I_620_) displayed a great variation from 0.22 to 18.3, with an 83-fold increase. Furthermore, the ratio of fluorescence intensity (I_505_/I_620_) exhibited a good linear relationship with NaHSO_3_ concentrations ranging from 0.5 μM to 50 μM (Figure 2b). The detection limit of B2P for SO_2_ derivatives was calculated to be 0.16 μM based on the recommendation of IUPAC (C_DL_ = 3S_b_/m).

The selectivity of B2P for sulfur dioxide over various interferents (200 μM), including biothiols (Cys, GSH and GSSG), amino acids (Ala, Arg, Lys and Glu) and representative ions (SO_4_^2−^, Cl^−^, NO_3_^−^ and Ca^2+^, Mg^2+^, Fe^3+^, Cu^2+^), was also tested. As shown in Figure 2c, only HSO_3_^−^ could trigger a significant change in the fluorescence intensity ratio (I_505_/I_620_) of the probe, while the other related species showed nearly no changes. Therefore, B2P had high selectivity for SO_2_.

### 3.3. Fluorescence Spectra of the Probe for B2P–SO_2_ Addition Adducts toward FA

After proving the suitability of the probe for detecting SO_2_, the reversibility of B2P–HSO_3_ (the adduct of the probe and SO_2_) was further investigated by adding FA. Results are shown in Figure 2d. With the addition of different concentrations of HCHO to the B2P–HSO_3_ (the product of 10 μM probe and 100 μM sodium bisulfite), the fluorescence emission at 620 nm was enhanced, and a significant fluorescence attenuation at 505 nm was observed. The fluorescence intensity ratio was saturated after the addition of 500 μM FA. The recovery of the spectral properties of the probe in the presence of aldehydes (acetaldehyde/MeCHO, propionaldehyde/EtCHO, n-butyraldehyde/PrCHO, 2-oxopropanal/AcCHO, benzaldehyde/PhCHO), other types of oxidizing substances (H_2_O_2_), biothiols (Cys, GSH and GSSG), amino acids (Ala, Arg, Lys and Glu) and representative ions (SO_4_^2−^, Cl^−^, NO_3_^−^ and Ca^2+^, Mg^2+^, Fe^3+^, Cu^2+^) was investigated. As can be seen in Figure 2e, FA was the most effective oxidant to restore the fluorescence intensity, whereas other oxidants, including H_2_O_2_ and other aldehydes, only exhibited a limited recovery rate. Furthermore, as shown in Figure 2f, the reversible sensing could be realized for at least five cycles.

### 3.4. Cellular Imaging of B2P

**Figure 3:**
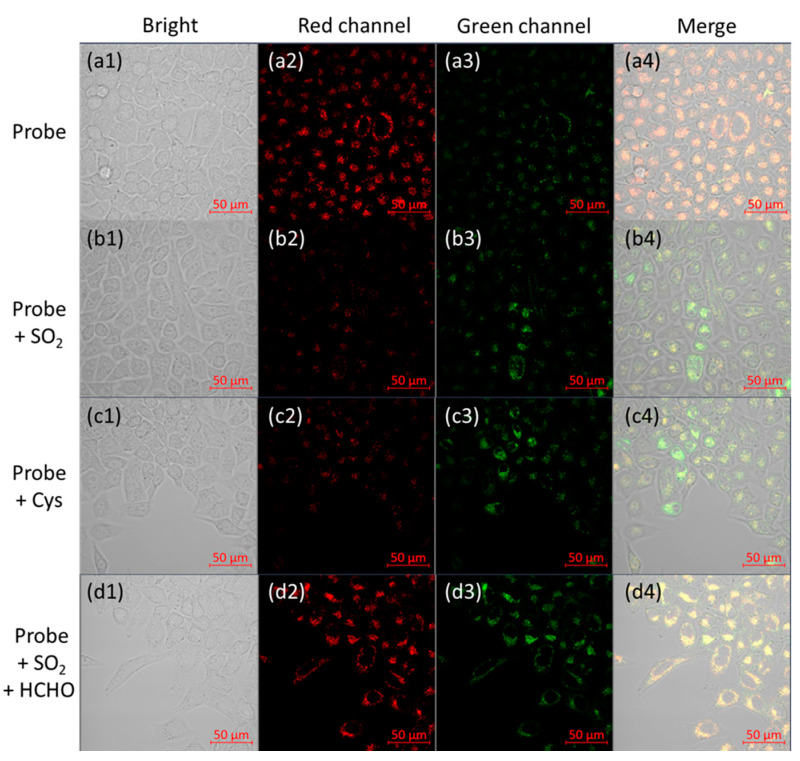
Confocal images of the probe’s responses to endogenous and exogenous SO_2_ and HCHO in HeLa tumor strain cells. (**a1**–**a4**) Cells were cultured with 10 μM B2P for 20 min; (**b1**–**b4**) cells were cultured with 10 μM B2P for 0.5 h then cultured with 50 μM of odium bisulfite for 20 min; (**c1**–**c4**) cells were first incubated with 10 μM B2P for 0.5 h, then incubated with sodium bisulfite (100 μM) for 20 min; (**d1**–**d4**) probe-loaded cells incubated with sodium bisulfite (50 μM) for 20 min were then incubated with HCHO (500 μM) for another 1 h. From left to right: bright field; red channel: λ_em_ = 580–650 nm; green channel: λ_em_ = 480–520 nm; merge. Scale bar, 50 μm.

Inspired by these satisfactory results, biological imaging experiments were carried out to confirm the feasibility of the probe to detect endogenous SO_2_ and FA. The ability of B2P to image reversible redox cycles of SO_2_ and FA in living cells was evaluated using HeLa cells. Firstly, HeLa cells were incubated with B2P at 37 °C for 20 min. Then, the cells to be imaged were washed with phosphate-buffered saline (pH 7.4) three times. According to Figure 3(a1–a4), cells cultured with B2P exhibited intense fluorescent signals in the red channel and relatively weak signals in the green channel. Upon the addition of sodium bisulfite (50 μM) and incubation for 20 min, the fluorescence signals in the red channel almost disappeared, and the green fluorescence increased (Figure 3(b1–b4)). Furthermore, in order to detect the endogenous SO_2_ produced by cells, Cys (500 μM) was added to the culture medium as a key contributor to intracellular SO_2_ production [39]. After 1 h of incubation, it could be observed with a confocal microscope that the fluorescence intensity in the red channel decreased, and the fluorescence intensity in the green channel increased; these results were similar to those for the addition of sulfur dioxide (Figure 3(c1–c4)). Additionally, when FA (500 μM) was further added into the dish of HeLa cells, already treated with B2P and HSO_3_^−^, for another 60 min, the intensity of the red fluorescence recovered, and the green fluorescence signal decreased (Figure 3(d1–d4)). Therefore, these results suggested that B2P was cell-permeable and capable of monitoring SO_2_ derivatives and FA in living cells.

### 3.5. Localization of the Probe in Cells

**Figure 4:**
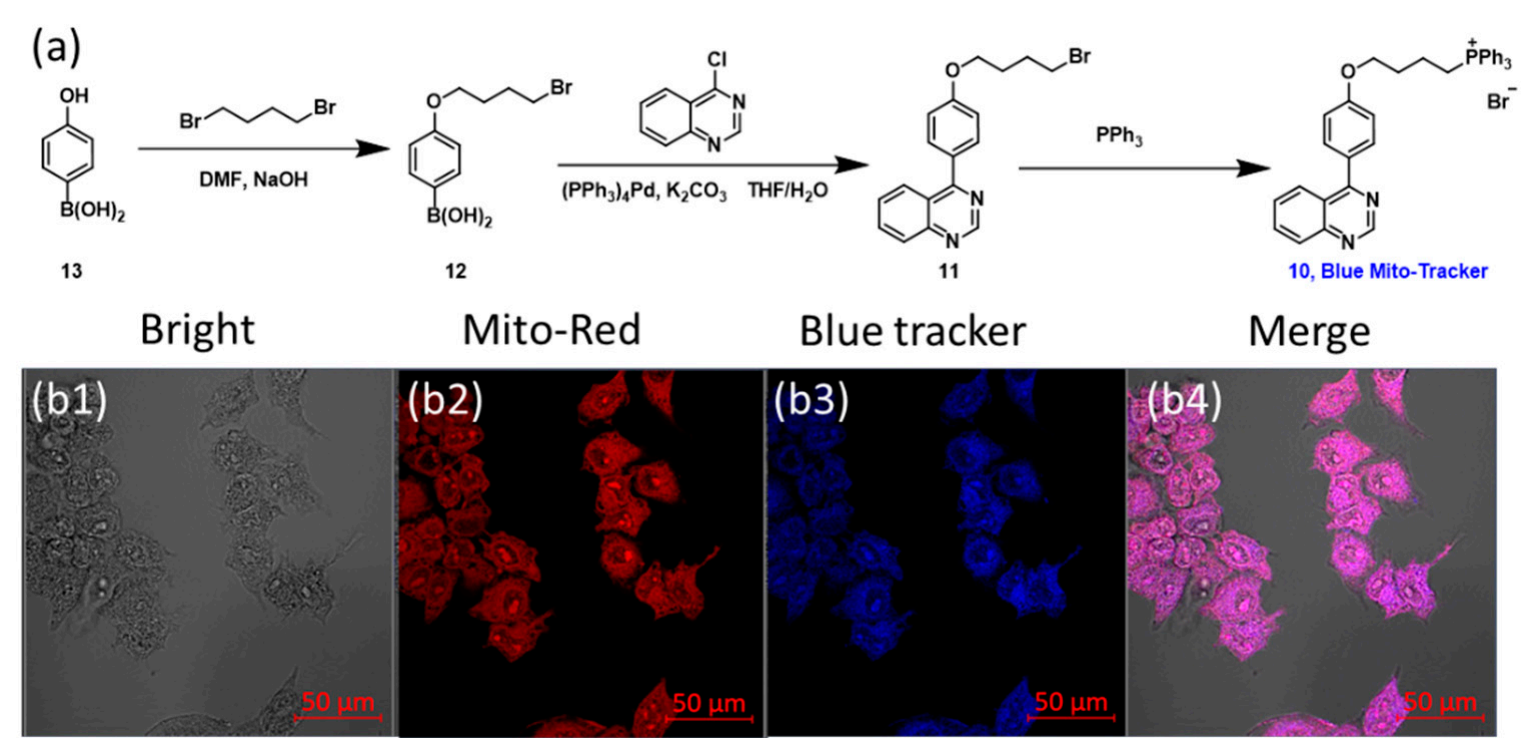
The synthesis of the self-made blue mitochondrial tracking dye (compound **10**) (**a**) and the colocalization effect of the self-made dye and commercially available MitoTracker Red (**b1**–**b4**). The Pearson’s R value of the red channel and blue channel was 0.91. Scale bar, 50 μm.

Considering that mitochondria are the metabolic centers of the sulfur elements [40,41,42,43] and redox centers [32] in cells, it is desirable to target the mitochondria in living cells with the probe. Therefore, the localization of the probe in cells was evaluated. However, due to the fact that the probe had obvious fluorescence emission in both red and green channels, the common red or green mitochondrial trackers could not be used for the colocalization analysis. Thus, a dye with blue fluorescence was designed and synthesized for cellular imaging experiments (Figure 4a). For detailed information, see the Supplementary Material (Figures S11–S15). To evaluate whether the new dye, showing blue fluorescence, could target mitochondria, HeLa cells were simultaneously incubated with MitoTracker Red (a widely used, commercially available mitochondrial tracker) and the newly synthesized blue tracker at 37 °C for 20 min. Fluorescence imaging results showed that the two dyes had excellent colocalization properties (Figure 4(b1–b4)). The Pearson’s coefficient R value of the red channel and blue channel was calculated to be 0.91.

**Figure 5:**
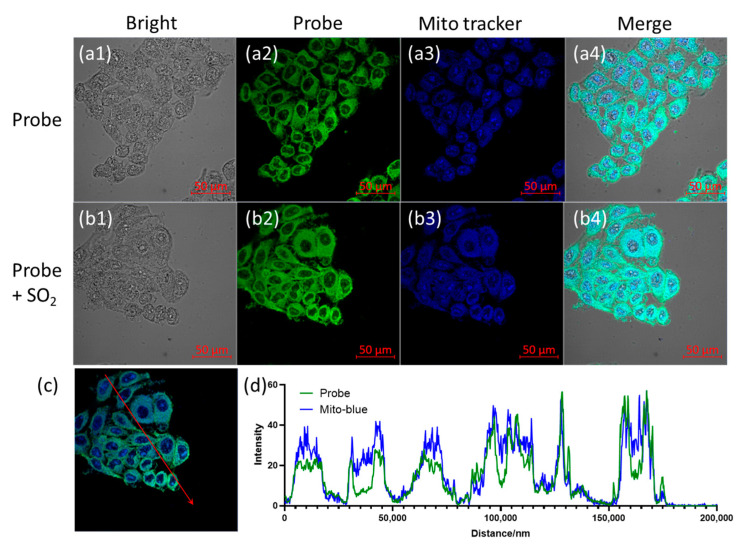
Confocal images of colocalization between the probe and newly synthesized blue mitochondrial tracker. (**a1**–**a4**) Cells were incubated with B2P and blue tracker (both 10 μM) for 20 min; (**b1**–**b4**) cells were incubated with B2P–HSO_3_ and blue tracker for 20 min. From left to right: bright field; green channel (for probe): λ_em_ = 480–520 nm; blue channel (for tracker): λ_em_ = 410–450 nm (λ_ex_ = 405 nm); merge. Scale bar, 50 μm. Furthermore, (**d**) shows the intensity of the green and blue fluorescence signal intensity on the red line in (**c**).

After confirming the effectiveness of the new blue tracer, we used it for the localization of probe B2P in living cells. After a coculture of the blue tracker and B2P, with cells at 37 °C for 20 min, confocal images were collected. As shown in Figure 4, no matter whether it was before (Figure 5(a1–a4)) or after the treatment with SO_2_ (Figure 5(b1–b4)), the probe had obvious colocalization with the blue tracker in cells. The corresponding Pearson’s coefficient R values, calculated with FIJI2 software, reached 0.82 and 0.76, respectively. Moreover, Figure 5d shows the intensity of the fluorescence signal on the red line in Figure 5c. It can be seen that the change in the relative intensity of blue and green fluorescence at different positions was always consistent. The above results confirmed that both B2P and B2P–HSO_3_ were located in the mitochondria of cells. This can be attributed to the fact that the probes carried positive charges in both forms and to the relatively high lipophilicity of the BODIPY molecule [43].

### 3.6. Live Zebrafish Imaging

**Figure 6:**
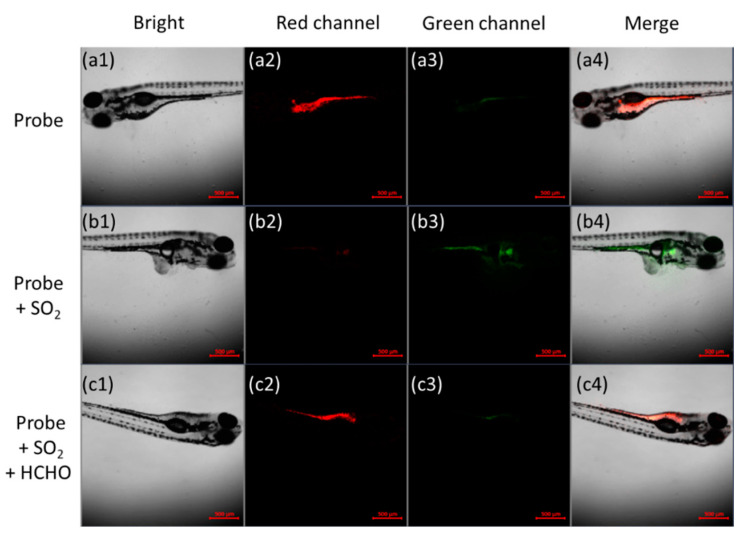
Confocal images of the probe in zebrafish. (**a1**–**a4**) Wild-type zebrafish were incubated with 20 μM B2P for 0.5 h; (**b1**–**b4**) zebrafish were incubated with 20 μM B2P for 0.5 h, then incubated with 100 μM sodium bisulfite for 0.5 h; (**c1**–**c4**) probe-loaded zebrafish were incubated with 100 μM sodium bisulfite for 0.5 h, and then cultured with 0.5 mM of HCHO for another 1 h. From left to right: bright field; red channel: λ_em_ = 580–650 nm; green channel: λ_em_ = 480–520 nm; merge; scale bar, 500 μm.

After successfully applying the probe to cells, the probe was applied to zebrafish. According to Figure 6(a1–a4), 5-day-old wild-type zebrafish fed with 20 μM B2P emitted a strong red fluorescence and weak green fluorescence. After treatment with sodium bisulfite, the fluorescence of the red channel in zebrafish almost completely disappeared, and the green fluorescence was significantly enhanced (Figure 6(b1–b4)). In contrast, as shown in Figure 6(c1–c4), when zebrafish were exposed to formaldehyde, the fluorescence in the red channel was restored, and the green fluorescence was weakened again. Therefore, the results showed that the probe could be successfully applied to monitor sulfur dioxide derivatives and HCHO in vivo.

## 4. Conclusions

Herein, we reported a ratiometric fluorescent probe based on an intramolecular FRET mechanism for the reversible detection of SO_2_ and FA. The probe B2P employed a benzopyranium moiety as the detecting site, which was highly reactive to sulfur dioxide and selectively reversed by FA. The BODIPY part, as an energy donor, also provided excellent optical properties for the probe. Thus, the probe achieved a high availability and selective ratio of fluorescence response for detecting SO_2_ and FA in vitro and in vivo. The introduction of a double cation structure not only enhanced the water solubility of the probe but also ensured that the prototype and adduct forms of the probe could be located in mitochondria, which was more conducive to the stability and repeatability of reversible detections. Meanwhile, the B2P had been proven to have eminent biosafety. The imaging experiments indicated that the B2P was capable of sensing concentration changes of sulfur dioxide and FA in living systems and could potentially be used for detecting redox balances in living organisms. The results demonstrated the diverse applications of the probe and suggested its potential for helping people to better understand the significant roles of SO_2_ and FA in redox processes in living systems.

## Data Availability

Not applicable.

## Supplementary Materials

The following supporting information can be downloaded at: https://www.mdpi.com/article/10.3390/bios12090715/s1, Figure S1, The biosafety test of the probe; Figure S2, The effect of pH value on fluorescence signal of the probe; Figure S3, Absorption spectra and emission spectra of compound 2 nad 3; Figure S4, The fluorescence emission spectra of the probe in different solvent; Figures S5–S15, NMR spectra of the compounds. Figure S16, MALDI-TOF mass spectrum of the probe.
