# Supplementary material for: Rational Construction of a Mitochondria-Targeted Reversible Fluorescent Probe with Intramolecular FRET for Ratiometric Monitoring Sulfur Dioxide and Formaldehyde
Source: Biosensors (Basel). 2022 Sep 3;12(9):715. doi: 10.3390/bios12090715 (PMC9496144; doi:10.3390/bios12090715)
Supplement: Supplementary file 1 [file biosensors-12-00715-s001.zip › biosensors-1800275-supplementary.pdf]

Rational Construction of a Mitochondria-targeted Reversible Fluorescent Probe with Intramolecular FRET for Ratiometric Monitoring Sulfur Dioxide and Formaldehyde

Contents

1. The biosafety test of the probe B2P

2. The effect of pH value on fluorescence signal of the probe

3. The synthesis of blue mitochondria tracker

4. Supplementary optical properties of the probe

5. NMR spectra of the probe and important intermediates

6. HRMS of the probe

1. The biosafety test of the probe B2P

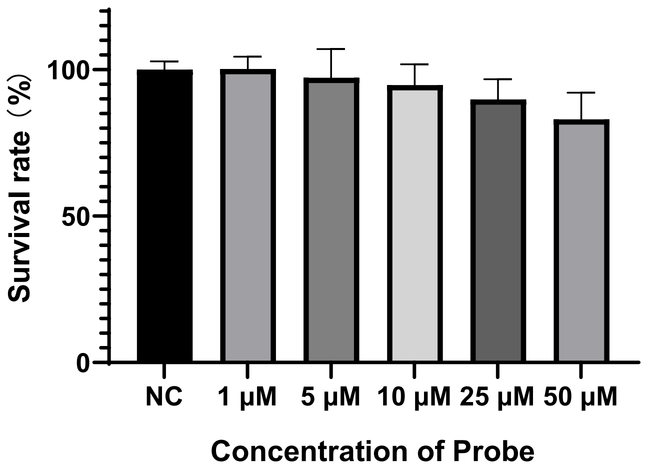

Figure S1. Cell survival rate after co culture with probe B2P for 12 hours. HeLa cells were cultured in 96 well plates with different concentration of the probe B2P for 12 h, and the survival rate was determined by Cell Counting Kit-8 (CCK-8).

2. The effect of pH value on fluorescence signal of the probe

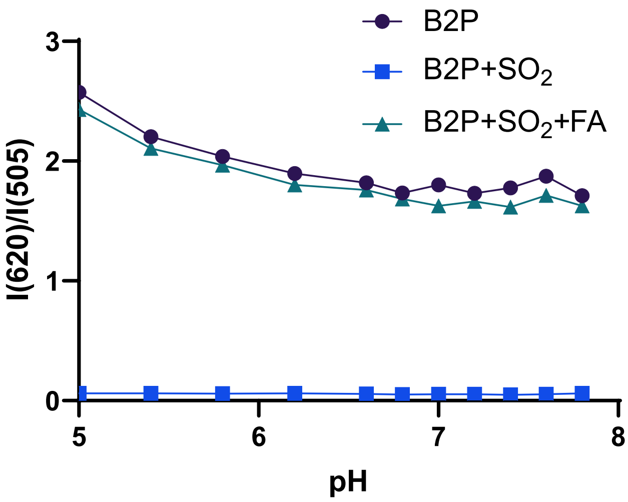

Figure S2. Fluorescence ratio (I(620)/I(505)) of the probe and the probe with analytes (SO_2 and FA) in different pH buffer.

3. The synthesis of blue mitochondria tracker

Compound 12:

To a dry flask with a stir bar, Compound 13 (138 mg, 1 mmol) dissolved in 15 mL DMF was added. Then, 40 mg NaOH (1 mmol) was added into the solution and the reaction was stirred at r.t. for 30 min, before 2.16 g dibromo butane (10 mmol) was added. The mixture was stirred overnight under nitrogen atmosphere. After TLC monitoring, 50 mL water was added and the reaction product was extracted by DCM (10 mL) 3 times. The combined organic layers were washed with saturated brine and dried over anhydrous sodium sulfate. The solvents were removed under reduced pressure. The residue was purified by silica column chromatography (DCM:MeOH, 20:1). 1H NMR (400 MHz, Chloroform-d) δ 8.25 – 8.13 (m, 2H), 7.09 – 6.98 (m, 2H), 4.11 (t, J = 6.0 Hz, 2H), 3.54 (t, J = 6.5 Hz, 2H), 2.12 (ddt, J = 13.6, 6.8, 1.7 Hz, 2H), 2.04 – 1.98 (m, 2H).

Compound 11:

Compound 11 was synthesized by a Suzuki coupling reaction. To a dry flask with a stir bar, Compound 12 (287 mg, 1 mmol), 4-chloro quinazoline (165 mg, 1 mmol) and $(PPh_3)_4Pd(0)$ (60 mg, 0.05 mmol) was added. Then 14 mL THF was added with a syringe under nitrogen atmosphere, before 6 mL 2M K_2CO_3 aqueous solution was added. The mixture was heated to 70 °C and stirred for 24 h. Then, 20 mL water was added and the reaction product was extracted by DCM (8 mL) 3 times. The combined organic layers were washed with saturated brine and dried over anhydrous sodium sulfate. The solvents were removed under reduced pressure. The residue was purified by silica column chromatography (DCM:MeOH, 30:1). 1H NMR (400 MHz, Acetone-

d6) δ 9.28 (s, 1H), 8.24 (dt, J = 8.5, 0.9 Hz, 1H), 8.08 (dd, J = 8.5, 1.3 Hz, 1H), 8.00 (ddd, J = 8.4, 6.7, 1.4 Hz, 1H), 7.88 – 7.81 (m, 2H), 7.73 (ddd, J = 8.3, 6.8, 1.3 Hz, 1H), 7.22 – 7.16 (m, 2H), 4.21 (t, J = 6.1 Hz, 2H), 3.64 (t, J = 6.6 Hz, 2H), 2.18 – 2.09 (m, 2H), 2.05 – 1.97 (m, 2H). ^{13}C NMR (101 MHz, $CDCl_3$) δ 175.58, 168.28, 154.43, 154.22, 152.69, 150.87, 150.79, 140.79, 140.55, 136.22, 133.90, 131.96, 130.21, 130.13, 128.73, 127.99, 127.88, 127.86, 127.67, 127.13, 123.14, 77.41, 77.09, 76.77, 72.44, 69.61, 33.77, 29.71, 29.66, 28.37, 28.32.

Compound 10 (The blue mitochondria tracker):

To a dry flask with a stir bar, a solution of Compound 11 (357 mg, 1 mmol) and PPh_3 (262 mg, 1 mmol) in 5 mL DMF was added. The mixture was heated to 100 °C under N_2 atmosphere, and stirred for 48 h. Then, 20 mL water was added and the reaction product was extracted by DCM (5 mL) 3 times. The combined organic layers were washed with saturated brine and dried over anhydrous sodium sulfate. The solvents were removed under reduced pressure. The residue was purified by silica column chromatography (DCM:MeOH, 15:1). 1H NMR (400 MHz, Chloroform-d) δ 9.32 (s, 1H), 8.17 (dd, J = 8.4, 1.3 Hz, 1H), 8.12 – 8.06 (m, 1H), 7.93 – 7.83 (m, 7H), 7.82 – 7.73 (m, 5H), 7.73 – 7.67 (m, 6H), 7.62 (ddd, J = 8.3, 6.9, 1.3 Hz, 1H), 7.08 – 7.01 (m, 2H), 4.24 (t, J = 5.6 Hz, 2H), 4.03 – 3.93 (m, 2H), 2.30 (p, J = 6.4 Hz, 2H), 1.92 (ddt, J = 15.7, 12.4, 6.0 Hz, 2H). ^{13}C NMR (101 MHz, $CDCl_3$) δ 167.79, 160.48, 154.56, 151.12, 135.04, 135.01, 133.77, 133.72, 133.67, 133.62, 131.72, 130.55, 130.43, 129.54, 128.79, 127.68, 127.20, 123.08, 118.73, 117.88, 114.70, 77.39, 77.07, 76.75, 66.71, 29.28, 29.11, 22.27, 21.77, 19.32, 19.28.

4. Supplementary optical properties of the probe

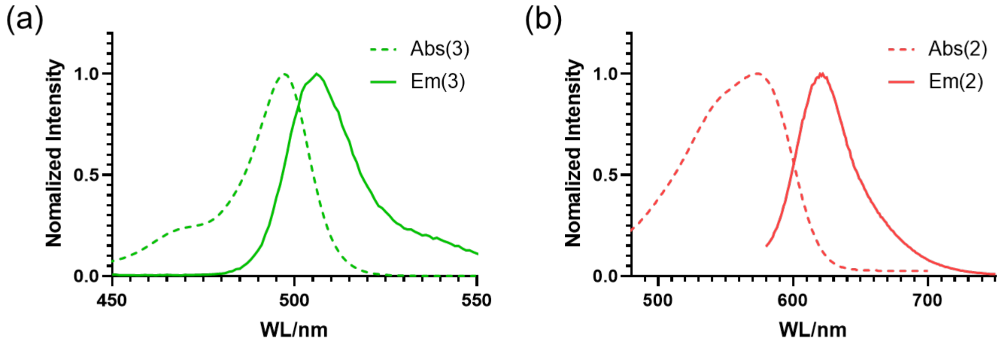

Figure S3. Absorption spectra and fluorescence emission spectra of compound 3 (a) and compound 2 (b).

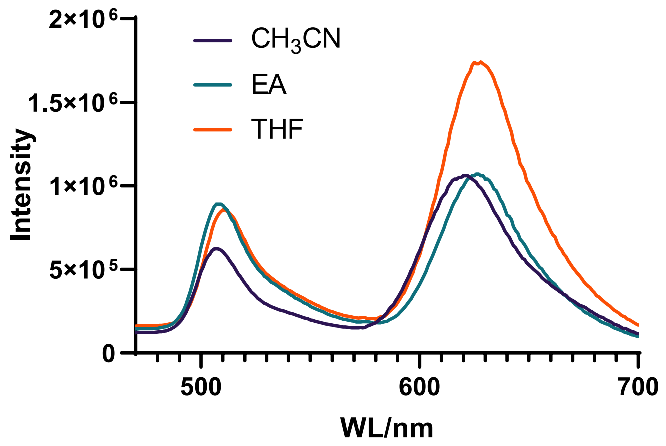

Figure S4. The fluorescence emission spectra of the probe in different solvent.

Table S1. Fluorescence quantum yield of probe.

QY /%	FL_{505}	FL_{620}
B2P	4.29	36.7
B2P-SO_3H	32.8	N.A.

QY: Quantum yield. NA: Not applicable. QY were acquired by using Horiba Flurolog TCSPC spectrometer with the quantum yield module.

5. NMR spectra of the probe and important intermediates

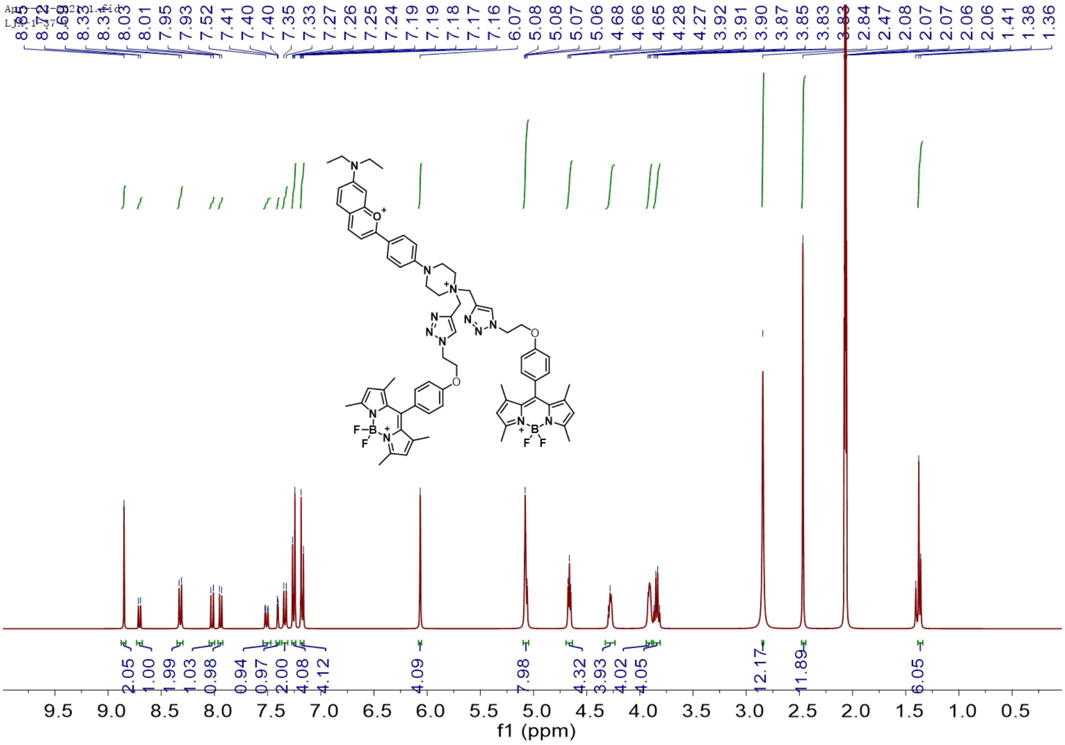

Figure S5. ^{1}H NMR spectrum of the probe B2P.

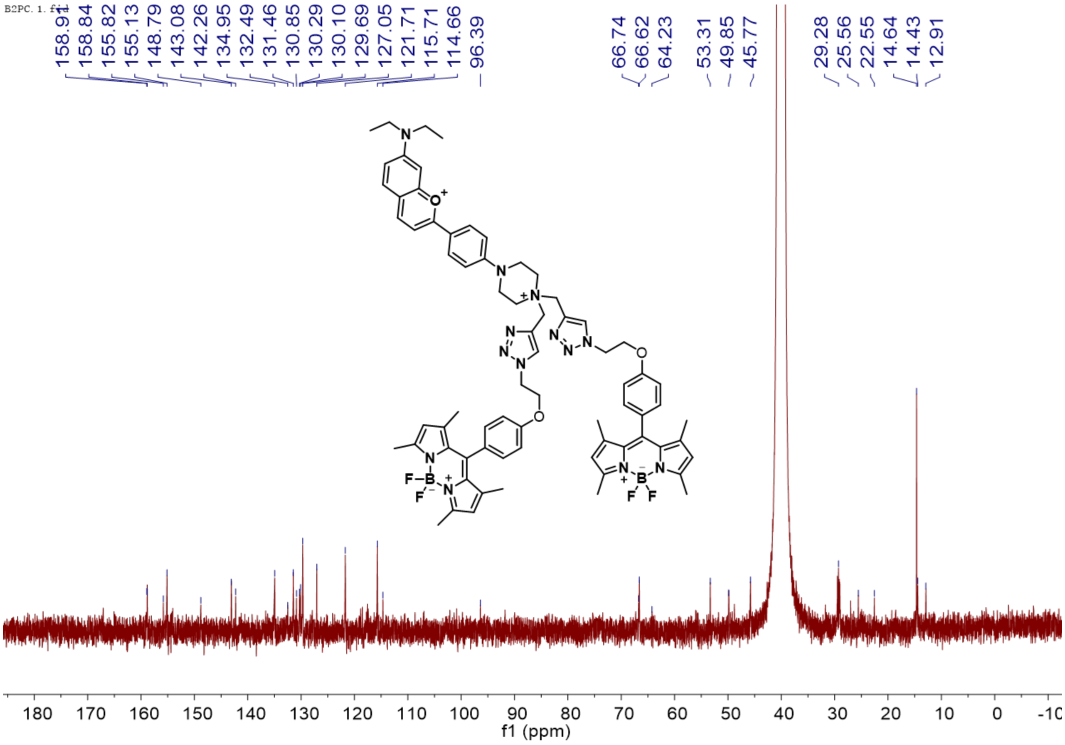

Figure S6. ^{13}C NMR spectrum of the probe B2P.

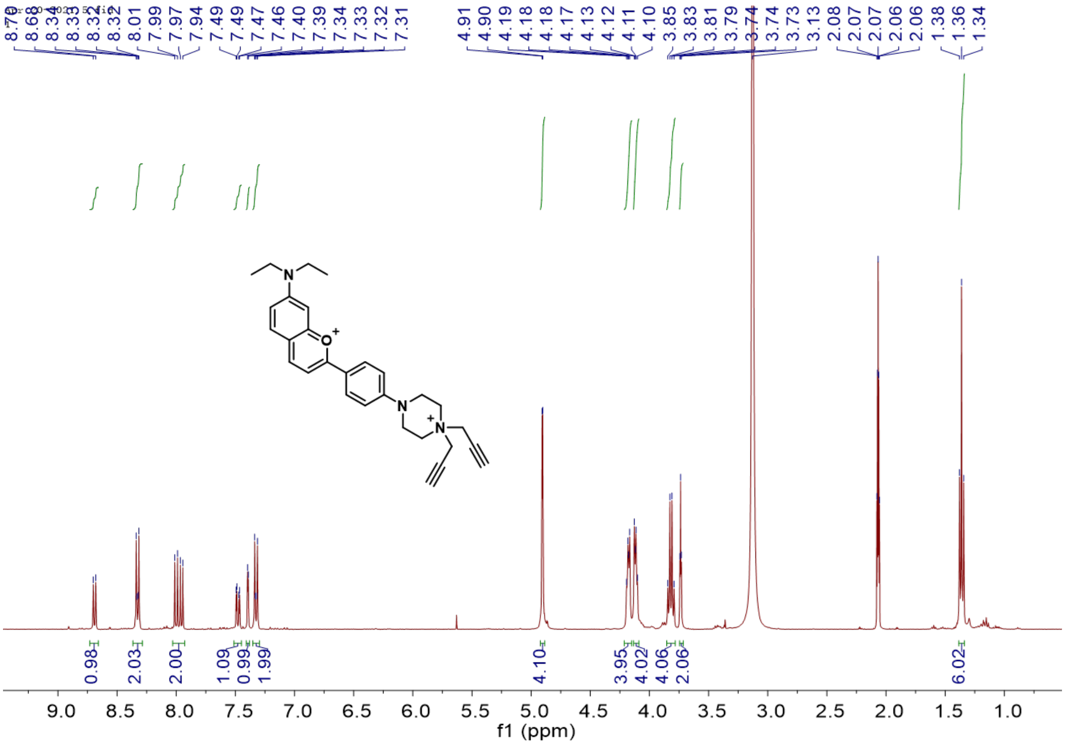

Figure S7. ^{1}H NMR spectrum of the compound 2.

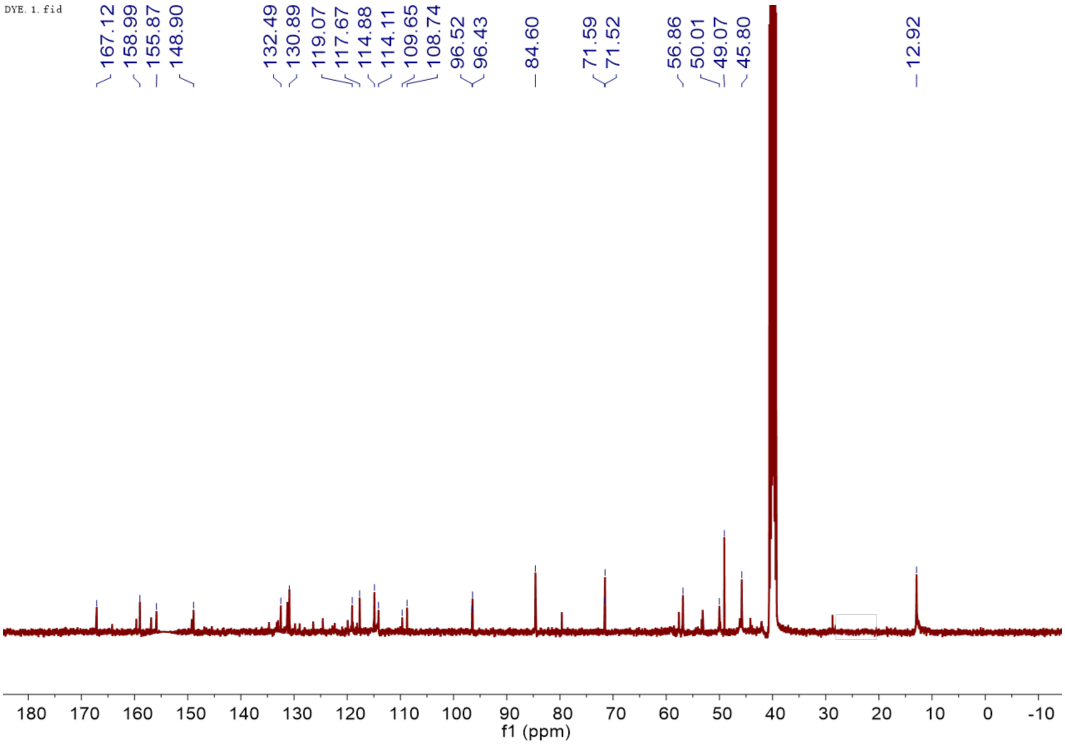

Figure S8. ^{13}C NMR spectrum of the compound 2.

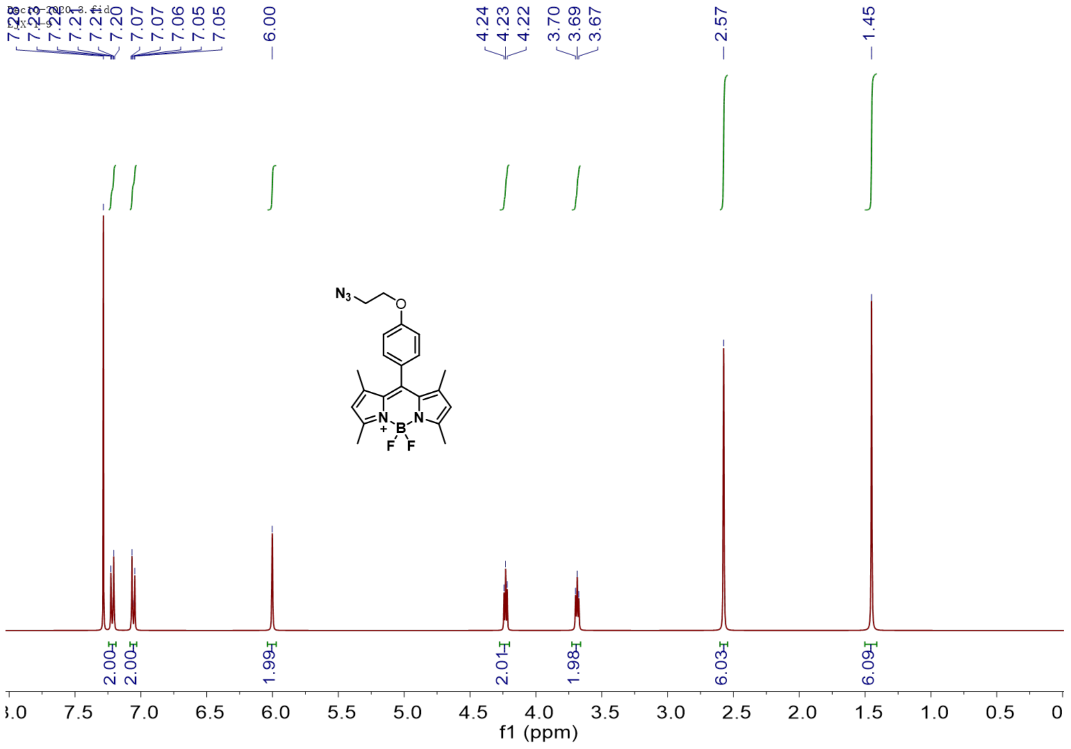

Figure S9. ^{1}H NMR spectrum of the compound 3.

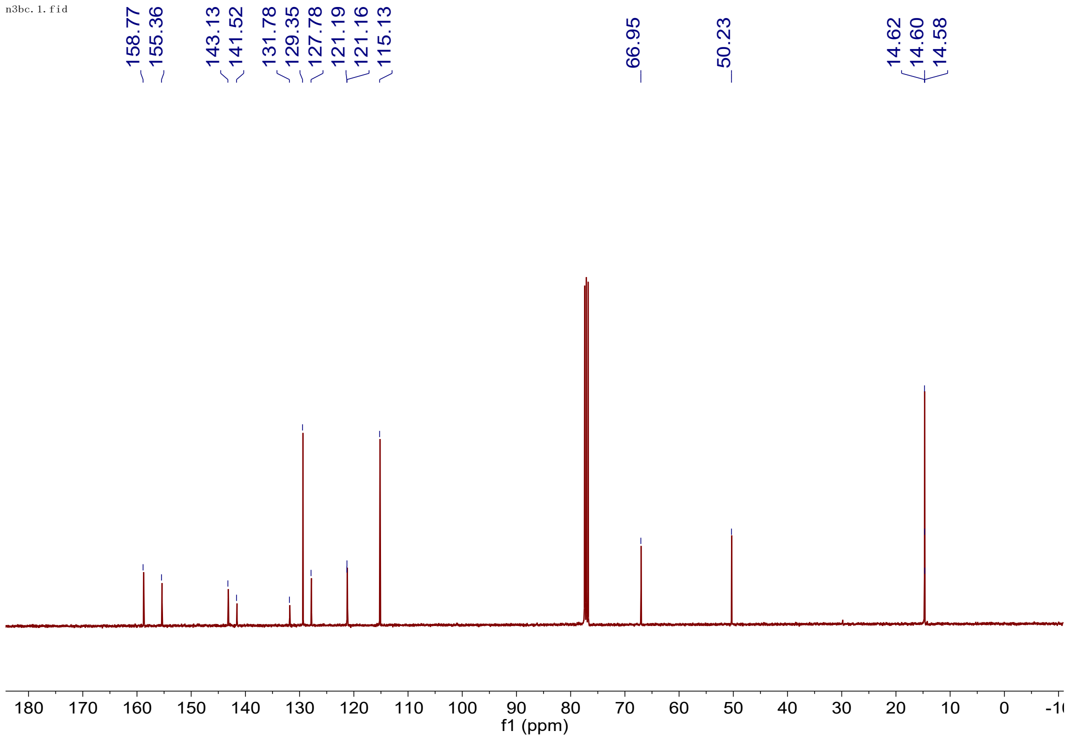

Figure S10. ^{13}C NMR spectrum of the compound 3.

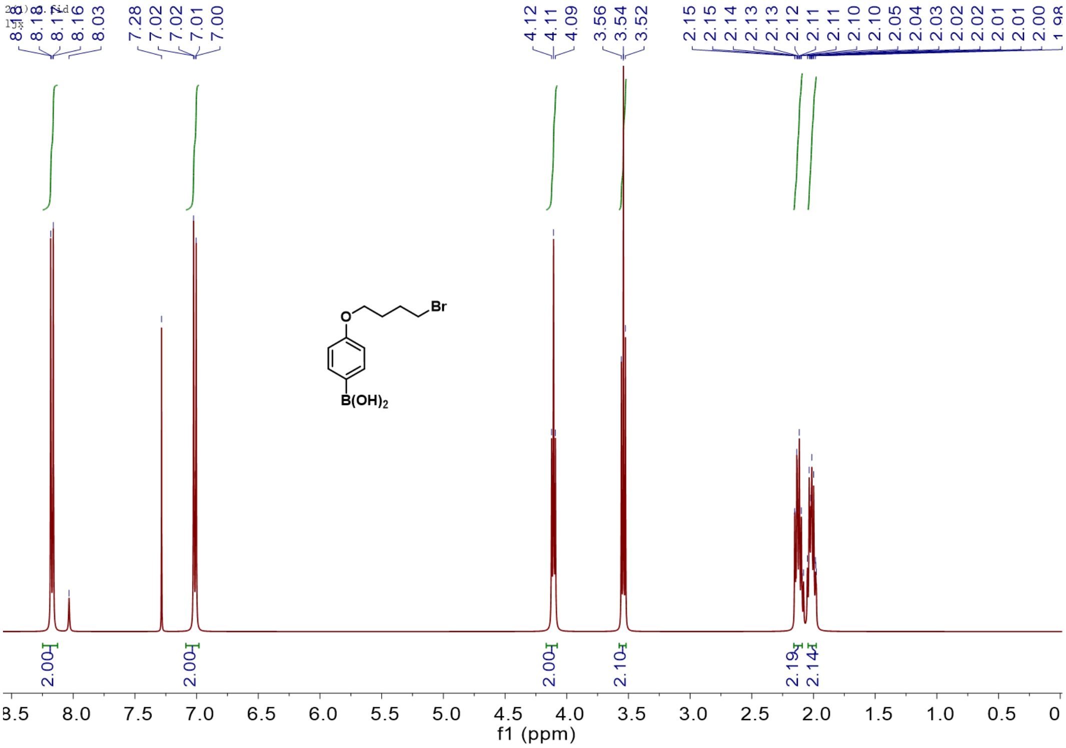

Figure S11. ^{1}H NMR spectrum of the compound 12.

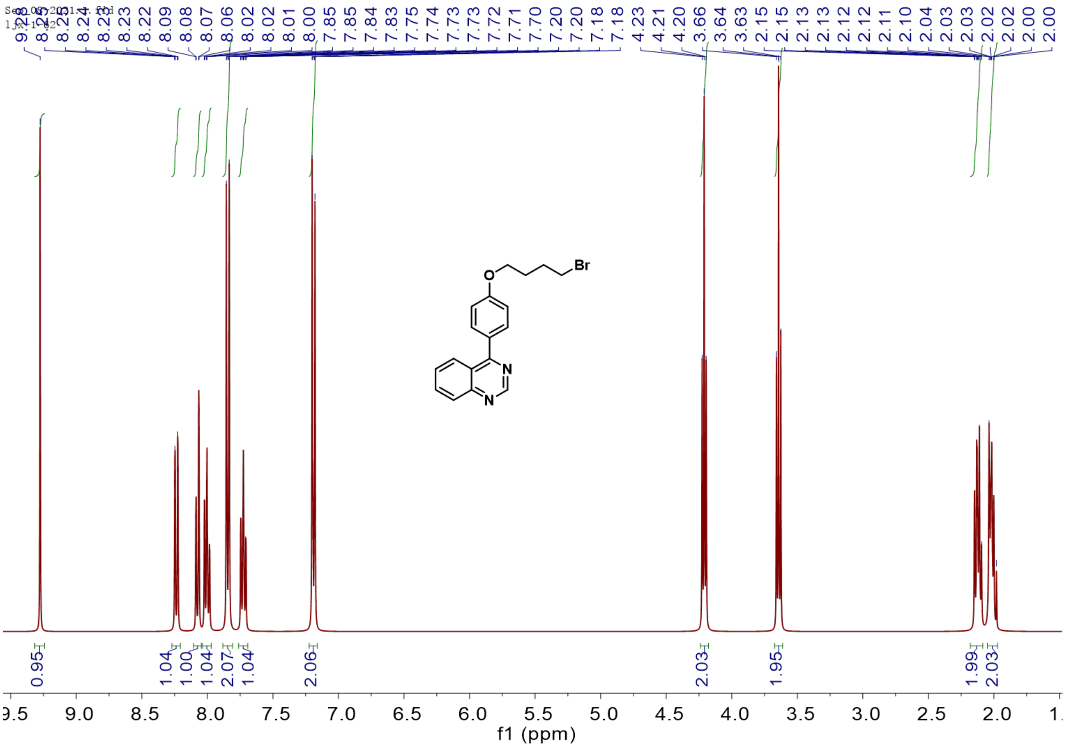

Figure S12. ^{1}H NMR spectrum of the compound 11.

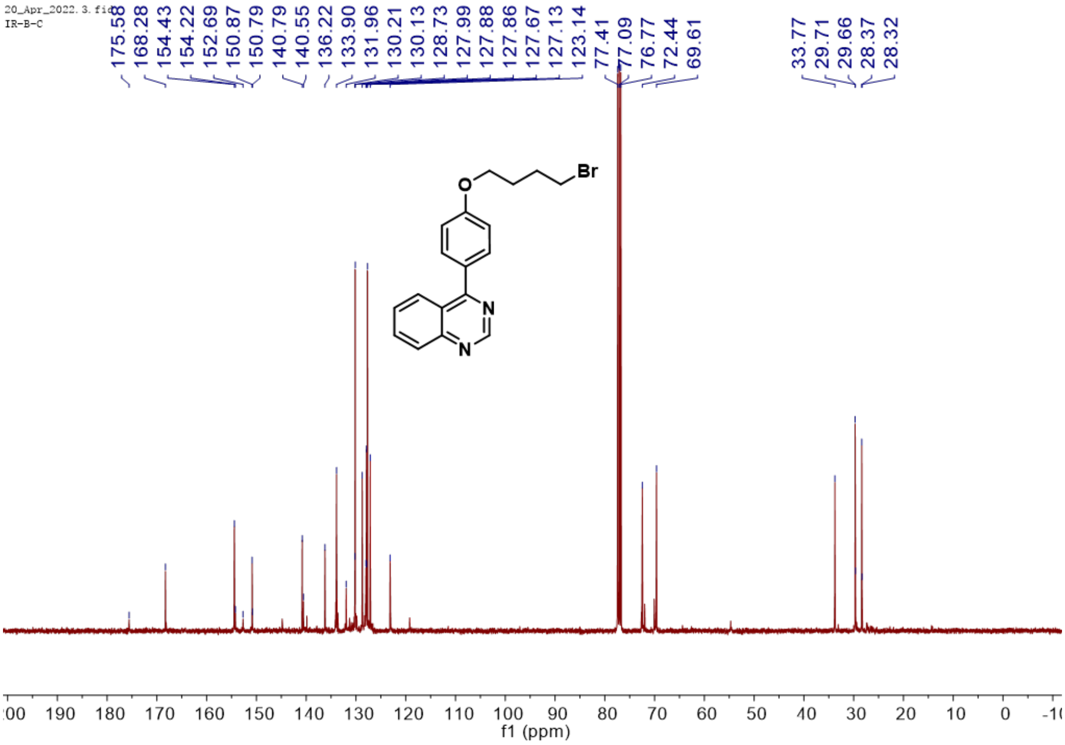

Figure S13. ^{13}C NMR spectrum of the compound 11.

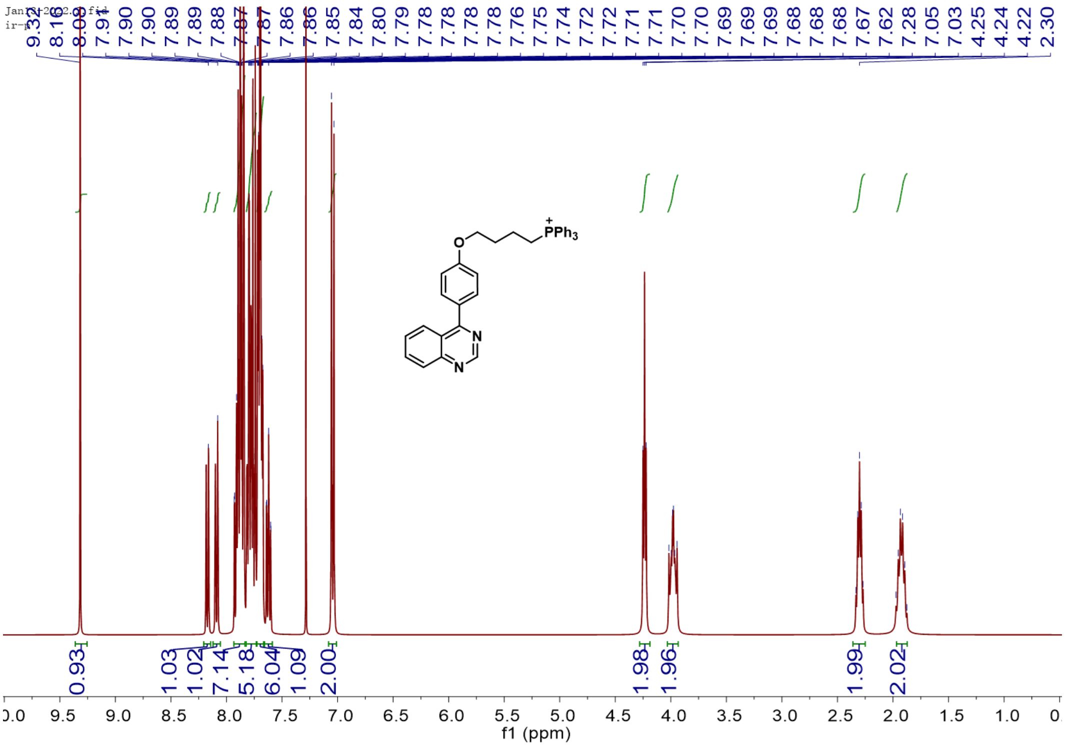

Figure S14. ^{1}H NMR spectrum of the compound 10 (blue mitochondria tracker).

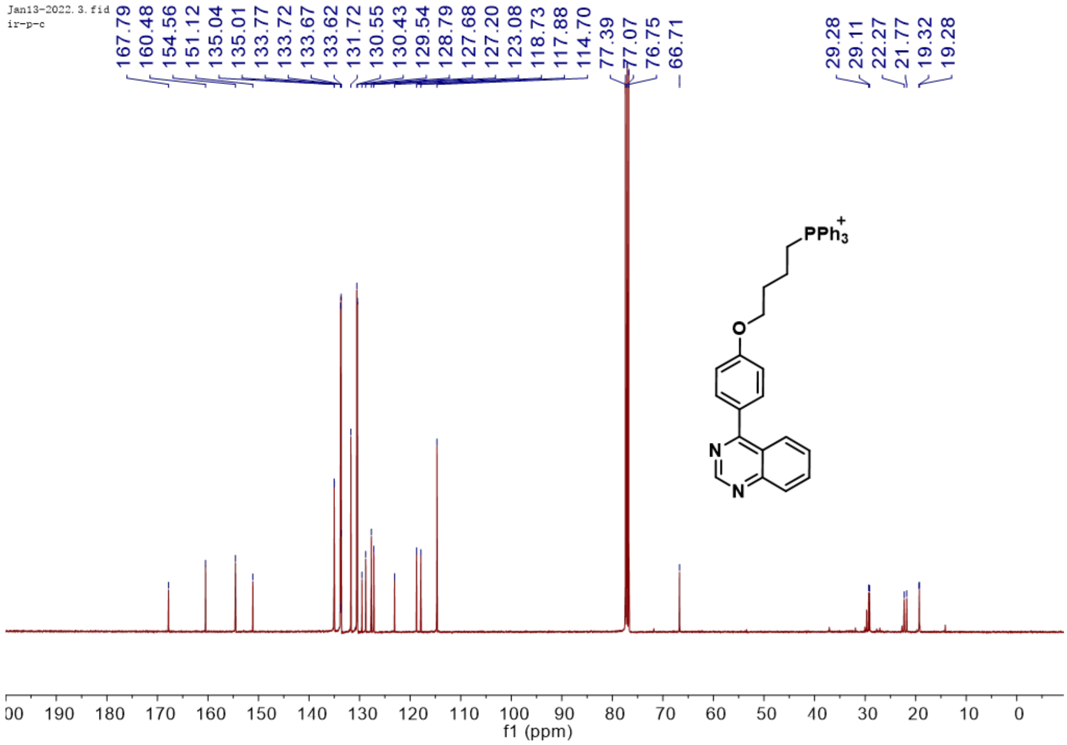

Figure S15. ^{13}C NMR spectrum of the compound 3.

6. HRMS of the probe

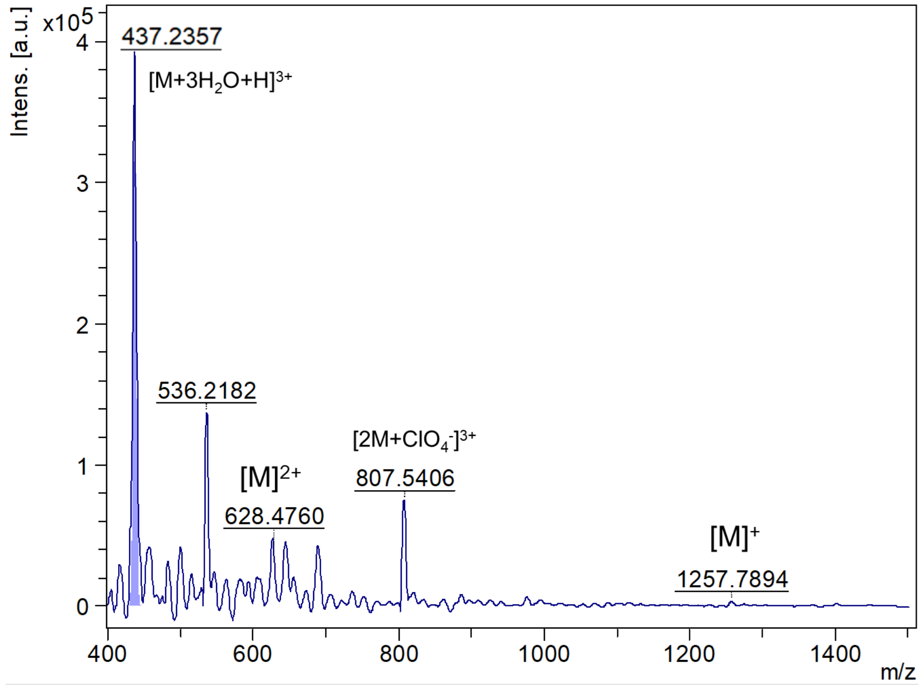

Figure S16.MALDI-TOF mass spectrum of the probe.
